# Is There a Need for Men's Health Training for Family Physicians in Canada?

**DOI:** 10.5402/2013/931265

**Published:** 2013-03-20

**Authors:** Andries Muller, Vivian R. Ramsden, Gill White

**Affiliations:** ^1^Academic Family Medicine, University of Saskatchewan, 3311 Fairlight Drive, Saskatoon, SK, Canada S7M 3Y5; ^2^Research Division, Department of Academic Family Medicine, University of Saskatchewan, West Winds Primary Health Centre, 3311 Fairlight Drive, Saskatoon, SK, Canada S7M 3Y5; ^3^College of Medicine, Regina General Hospital, 1440-14th Avenue, Regina, SK, Canada S4P 0W5

## Abstract

*Objective*. The goal of this study was to explore which topics were rendered important to incorporate into a men's health curriculum for family medicine resident training. *Design*. A mixed-methodology was used. A case study method with a sequential transformative strategy was utilized. A quantitative survey was sent to the 17 program directors of Canadian family medicine training programs. This was followed by a qualitative phase with interviews of selected program directors and two focus groups with practicing family physicians from a rural and an urban clinic. *Main Findings*. Certain issues were identified for incorporation into a men's health curriculum for family medicine resident training. These issues were grouped in three groups: male sexual and reproductive health, general topics, and procedures specific to men's health. *Conclusion*. It appears that there is no formal curriculum to address any of these issues in any of the current family medicine training programs in Canada. Based on the information gathered from participants in this study, there is a great need for such a curriculum to exist.

## 1. Introduction

The Men in Australia Telephone Survey (MATeS) was conducted in 2003 and is still the only whole-of-nation, population-based study focusing on the reproductive health and other issues of middle-aged and older Australian men [1]. Two of the main outcomes of this survey were the fact that a significant number of men are affected by reproductive health disorders (e.g., one in three men over the age of 40 years suffers from erectile dysfunction) and that men are indeed interested and concerned about their health. Almost 90% of the men in this study over the age of 40 years had consulted their physicians in the previous 12 months.

A study by White and Holmes again showed that men die earlier than women and they revealed this to be true in all 44 countries that they used in their sample [2]. It is therefore important that family physicians possess the necessary skills in dealing with the health care of their male patients. But what are these skills that are expected of a family physician in order to be “competent” in caring for male patients? This study sets out to do just that.

The goal of this study was to explore the various issues that program directors of family medicine training programs and practicing family physicians express as important and therefore should be included in a men's health curriculum for family medicine residency training programs.

## 2. Methods

A mixed-methodology approach, using both qualitative and quantitative methods, was used, specifically a sequential transformative design [3]. In this form of research design, which has two distinct data collection phases, one follows the other and builds on the earlier phase. Mixed-methodology study design was chosen because it was anticipated that a relatively low number of participants would be recruited for the various components of the study. This study design will also compensate for the fact that early saturation might (and in fact did) occur in the interview section. 

In this study, a quantitative survey was sent electronically to all program directors and site directors of the seventeen family medicine residency training programs in Canada. This was followed by a qualitative phase where interviews were arranged with some of the program directors and site directors. A matrix was created to purposefully select participants that have a men's health curriculum as well as some that did not. However none of the participants stated that they had a men's health rotation or curriculum. Participants were therefore selected based on availability and the quality of information received on the surveys. Saturation of information was felt to have occurred after only three interviews as no new information was obtained. Two focus groups were also conducted. One group consisted of four urban family physicians and the other of four rural family physicians, one resident, and one medical student. It was not the plan of the researcher to have a resident and medical student in the focus group, but they were working with the physicians at the time of the interview and felt that it would have been a good experience for them. It turned out that it was also a valuable source of information for the researcher, and therefore their comments were included in the analysis.

A certificate of approval was received from the University of Saskatchewan's Behavioural Research Ethics Board prior to starting this research endeavour. 

Many elements of the questionnaire focussed on different topics that might be considered relevant to men's health and whether the participants believed that it was important to be included in a men's health curriculum. The topics were chosen based on an extensive literature review. The participants were asked to select from each group all the topics they considered important to have in a men's health curriculum. 

The topics were grouped as follows:

1. male sexual and reproductive health (e.g., prostate cancer, erectile dysfunction, etc.),
2. general health relevant to men (e.g., cardiovascular disease in men, alcoholism, etc.),
3. procedures specific to men's health (e.g., vasectomies, circumcisions, etc.),

Participants were then asked to rank their top ten choices in the first group and their top five choices in the other two groups. The difference in ranking items is due to the difference in the number of items in each of the lists. Points were allocated to their ranked lists, for example if a topic was ranked first, it was scored 10 points in a rank list of ten, second was scored 9, and so on. Means, medians, and modes were calculated for all the responses. The topics were initially sorted in order of importance by using the means and secondly by the median. At the end of the survey, participants were invited to add any topic(s) that they believed should be included in a men's health curriculum for family medicine residency training programs. 

A case-based approach was used for the qualitative portion of the research. Cases can come in a variety of forms. In this study individual cases (interviews with program directors) as well as group cases (Focus groups) were used [4]. The responses from the quantitative questionnaire formed the basis of the semistructured interviews that were conducted with selected program directors. Some aspects of the questionnaire were used to prompt discussion in the focus groups. 

The discussions within the focus groups were recorded with both audio and video recording equipment and later transcribed. (Videotaping was used only to identify different speakers during transcription.) Interviews with program directors were only voice recorded.

These interviews and focus groups were transcribed by the researcher and imported into the NVivo (Version 9) software [5]. Each interview and each focus group were treated as an individual case.

Through deductive reasoning, certain themes were identified from the questionnaire data. This implies that general statements were synthesised into more specific statements. Some of the specific statements were also grouped in “themes” that belong together. These predetermined themes were registered as “nodes” in the NVivo software and were used while analysing the qualitative data. Further themes (or nodes) evolved as the transcriptions were analysed.

Although both the qualitative and quantitative aspects of the study had questions about past and current training in men's health, this paper will focus on what participants think that the content of a men's health curriculum for family medicine residency training should contain.

The author was involved in all aspects of this project, including conducting the interviews and focus groups as well as transcribing the interviews and analyzing the data.

## 3. Findings

### 3.1. Quantitative Data

A total of eleven questionnaires, which represented 10 (59%) of the 17 family medicine residency training programs in Canada, were completed and returned. In one case, two different sites in the same program returned a survey. Even though the number of responses might appear low, it is still considered above expected when taken into consideration the overwhelming number of surveys that ends up on program directors' desks. The rank list of problems identified from group one (sexual and reproductive health) is represented in Table 1.  Table 2 represents the rank list for topics related to general health and Table 3 presents the rank list for procedures. No new topics were added by any of the participants in the quantitative section of the study.

### 3.2. Qualitative Data

**“Men are considered to be rather uncomplicated, you know. They die early but no one seems to care”—program director.**

*Theme 1. Need for Men's Health Curriculum.* Following are some of the quotes from Program Directors and practicing physicians regarding a men's health curriculum in Family Medicine Residency Training Programs.

**“We have women's health. There should be something in men's health—it just makes common sense”—focus group participant.**

**“Everybody thinks it [men's health] equals erectile dysfunction… it's way broader…”—focus group participant.**

*Theme 2. Topics to Include in a Men's Health Curriculum.* The participants were asked to think of different topics that they would like to see in a men's health curriculum. Most of the topics that were listed in the quantitative questionnaire were mentioned again in the interviews and focus groups. Some of the other topics that were discussed are mentioned in the following quotes.

**“Screening guidelines for different age groups”—focus group participant.**

**“Issues around men who have sex with men…”—focus group participant.**

**“Talk about the Gardisil vaccine…”—focus group participant.**

**“I think mental health issues for men are really lacking”—focus group participant.**

Mental health issues came up in several of the interviews and focus groups. Even though “Psychiatry” was one of the topics in the quantitative survey, the participants in the focus groups and interviews wanted suicide to be singled out as an important topic in men:

**“Single elderly male has much higher rate of suicide”—focus group participant**

Some physical conditions were also mentioned.

**“Development of osteoporosis in men”—focus group participant.**

**“Semen analysis interpretation…male infertility”—focus group participant.**

Even though the Program Directors did not rank it very high, participants in the focus groups reported that they would have liked to be more competent in dealing with priapism and Peyronie's disease.

When it came to procedures, no new procedures were mentioned that had not already been covered in the survey responses of the program directors. Two of the participants in the focus groups did however classify digital rectal exams as a “procedure” and would have liked more and better formal training in performing this correctly.

*Theme 3. Structure of a Men's Health Curriculum.* The focus groups were asked how they would have preferred to learn about men's health issues as a student or a resident. The idea of a men's health clinic, preferably in an academic setting, was discussed and received favorably by other participants of the urban focus group:

**“…involve men of all over the city to come in as patients…and so do a rotation in such a clinic. I think it's a great idea”—focus group participant.**

## 4. Discussion

The majority of the top ten topics listed in Table 1 did not come as a huge surprise. These were topics that most family physicians deal with on a daily basis. The only topic that ranked higher than expected was that of andropause or, as it is now known, late onset hypogonadism (LOH) [6]. This could be due to the fact that several new testosterone replacement products became available in Canada in the last few years and some media attention has been directed that way. Family physicians might therefore feel some pressure in becoming competent regarding this issue.

Even though some conditions in Table 1 have not been ranked at all by program directors, it does not mean that it is not important. In hindsight, it might have been interesting to have given the same lists to members of the focus groups to complete and see how (if at all) it differs from the ranking of the program directors.

With the rank list of topics listed in Table 2, it was interesting to note how high nonphysical issues were ranked. One would expect that cardiovascular disease in men would be high on the list since it remains one of the highest causes of mortality in men (second only to cancer) [7]. Instead the mental health or psychiatric conditions such as abuse, alcoholism, and general psychiatry were prominent.

The list of procedures that were ranked in Table 3 held no surprises at all. The majority of these procedures form part of the core procedure list that is used in most family medicine residency training programs in Canada [8]. These are procedures that family medicine residents expect to be taught and that the general public expect that family physicians will be able to perform.

Topics that were identified and discussed in the interviews and focus groups reflect the variety of issues that family physicians deal with on a regular basis. It was also clear that the participants (mostly practicing physicians) did not feel comfortable dealing with issues related to men's health. Many of the participants told stories about how they learned many of these skills after being in practice for a while. If family physicians can become competent in these skills before they start their practice, this could cut down on many unnecessary and costly referrals to specialists.

The topics that were identified as important to incorporate into a men's health curriculum compare well to curricula developed in the UK, Australia, and the State of Hawaii [9–11]. There are many topics that are common to other curricula, but not all of them are unique to men's health. If one only focuses on male-specific issues, the American Academy of Family Physicians also identified male baldness as a unique and separate topic in their curriculum [12]. The College of Family Physicians of Canada has goals and objectives for general care of the adult in their standards for accreditation [13]. It is however essential to separate the unique issues related to men's health into a separate curriculum (as is the case with women's health) in order for it to receive the importance it warrants.

## 5. Limitations

This study was performed in a Canadian context and can therefore not neccesarily be extrapolated to other countries. The sample size in each of the different components is quite low due to the small number of possible participants. This has been addressed in the methodology section. Some of the surveys contained the minimum required answers and did not contain any further ideas. It would have been helpful if participants could have added information that was not part of the questionnaires. The fact that the principle author was the only person conducting the interviews and analyzing the data could be seen as a limitation. This was unfortunately an expectation as this study formed part of an Ph.D. research program.

## 6. Conclusion

Participants in both the quantitative and qualitative aspects of the study provided lists of men's health issues that could and should be seen as competencies that family medicine residents attain during their training. It appears that there is no formal curriculum to address any of these issues in any of the current family medicine residency training programs in Canada. Based on the information gathered from participants in this study, a common theme emerged in that there is a great need for such a curriculum. This curriculum has to comply and mesh with the Triple C curriculum as proposed by the College of Family Physicians of Canada [14].

## Figures and Tables

**Table 1:** List of male sexual and reproductive health topics identified by program directors in order of importance.

Rank	Topic	Mean	Median
1	Benign Prostatic Hypertrophy	9.09	9
2	Prostate cancer	8.64	10
3	Erectile dysfunction	7.64	8
4	Sexually Transmitted Infections	5.55	6
5	Prostatitis (acute)	3.36	5
6	Testicular cancer	3.27	3
7	Epididymitis	2.36	2
8	Andropause	1.94	0
9	Prostatitis (chronic)	1.82	2
10	Testicular torsion	1.82	0
11	Contraception for men	1.82	0
12	Urinary Tract Infections in men	1.45	0
13	Undescended testes	1.36	0
14	Hydrocele	0.73	0
15	Phimosis	0.27	0
16	Varicocele	0.27	0
17	Balanitis	0.27	0
18	Male infertility	0.18	0
19	Premature ejaculation	0.09	0
20	Gynecomastia	0.09	0
21	Peyronie's disease	0	0
22	Penile cancer	0	0
23	Paraphimosis	0	0
24	Other sexual dysfunction	0	0
25	Priapism	0	0
26	Genital trauma	0	0
27	Klinefelter disease	0	0

**Table 2:** List of topics related to general health identified by program directors in order of importance.

Rank	Topic	Mean	Median
1	Alcoholism	3.00	3
2	Abuse	3.00	2
3	Psychiatry	2.82	3
4	Eyes and Ear Nose and Throat	2.73	0
5	Cardiovascular	2.27	1
6	Pharmacology	2.09	0
7	Respiratory	1.82	0
8	Musculoskeletal	1.55	0
9	Skin	1.45	0
10	Gastrointestinal	1.00	0
11	Neurological	0.36	0

**Table 3:** List of procedures identified by program directors in order of importance.

Rank	Topic	Mean	Median
1	Newborn circumcision	1.36	1
2	Reduce paraphimosis	1.36	0
3	Vasectomy	1.18	0
4	Drain hydrocoele	1.00	0
5	Bladder catheterization	0.91	1
6	Intracavernosal injections	0.82	0
7	Adult circumcision	0	0
8	Prostate biopsy	0	0
